# Laparoscopic liver resection as a safe and efficacious alternative to open resection for colorectal liver metastasis: a meta-analysis

**DOI:** 10.1186/1471-2482-13-44

**Published:** 2013-10-01

**Authors:** Yanming Zhou, Yaqing Xiao, Lupeng Wu, Bin Li, Hua Li

**Affiliations:** 1 Department of Hepatobiliary & Pancreatovascular Surgery, Oncologic Center of Xiamen, First affiliated Hospital of Xiamen University, Xiamen, China; 2 Department of Digestive Diseases, First affiliated Hospital of Xiamen University, Xiamen, China

**Keywords:** Laparoscopic liver resection, Colorectal liver metastasis, Meta-analysis

## Abstract

**Background:**

The safety and efficacy of laparoscopic liver resection (LLR) for colorectal liver metastasis (CLM) remain to be established. A meta-analysis was undertaken to compare LLR and open liver resection (OLR) for CLM with respect to surgical and oncologic outcomes.

**Methods:**

An electronic search was performed to retrieve all relevant articles published in the English language by the end of March 2013. Data were analyzed using Review Manager version 5.0.

**Results:**

A total of 8 nonrandomized controlled studies with 695 subjects were analyzsed. Intra-operative blood loss, the proportion of patients requiring blood transfusion, morbidity and the length of hospital stay were all significantly reduced after LLR. Postoperative recurrence, 5-year overall and disease-free survivals were comparable between two groups.

**Conclusions:**

LLR for CLM is safe and efficacious. It improves surgical outcomes and uncompromises oncologic outcomes as compared with OLR.

## Background

Colorectal cancer (CRC) is among the most frequently encountered malignant neoplasms in the world. Approximately 50% CRC patients developed liver metastasis during disease evolution, which is a major cause of cancer death [1]. Conventional open liver resection (OLR) is an effective treatment for colorectal liver metastasis (CLM), offering a 5-year survival of 16-74% and a 10-year survival of 9-69% [2].

Lparoscopic liver resection (LLR) was first reported as a minimally invasive procedure two decades ago [3], but many surgeons are reluctant to accept it as an alternative to an open approach due to the potential risks of intraoperative complications and oncologic adequacy. With tremendous instrumental and technological advances in the laparoscopic field in recent years, increased numbers of reports on LLR have been published [4]. A number of case-series reports and comparative trials have demonstrated the potential benefits, safety and feasibility of LLR for CLM [5-16]. However, no related evidence has been reviewed systematically. In accordance with recommendations of the Preferred Reporting Items for Systematic Reviews and Meta-Analyses (PRISMA) Statement [17], we made a meta-analysis to provide a better quality of evidence in the literature to support the recommendation of LLR as an alternative option for CLM treatment.

## Methods

### Study selection and data extraction

A MEDLINE, EMBASE, OVID, and Cochrane database were searched to identify all clinical trials published as full papers in the English language that compared LLR and OLR for CLM between July 1992 and March 2013. The following Mesh search headings were used: "laparoscopic liver resection", "colorectal cancer", and "colorectal liver metastases". The bibliographies of relevant articles were reviewed manually to identify additional trials.

The following parameters were extracted from each study and tabulated by two investigators (BL and LPW) independently: name of first authors, year of publication, study design, number of patients in each arm, patient baseline characteristics, and outcomes of interest. All relevant texts, tables and figures were reviewed for data extraction. If the study provided medians and interquartile ranges instead of means and SDs, the means and SDs were imputed as described by Hozo *et al*. [18]. Discrepancies between the reviewers were resolved by discussion and consensus.

### Outcomes of interests

Surgical outcomes: operative parameters (duration of operation, intra-operative blood loss, and need for blood transfusion), postperative adverse events (morbidity and mortality rates), and postoperative recovery (hospital stay, time to bowel movement, time to oral intake, and requirement for analgesia).

Oncologic outcomes: resection margin, recurrence, 5-year overall survival (OS) and disease-free survival (DFS).

### Criteria for inclusion and exclusion

To be included in the analysis, a study had to compare LLR and OLR for CLM. If dual (or multiple) studies were reported by the same institution, only the most recent was used. Unpublished studies, abstracts, letters, proceedings from scientific meetings, editorials and expert opinions, reviews without original data, case reports and studies lacking control groups were excluded. Trials that involved heterogeneous groups of patients with a variety of hepatic disesase were also excluded.

### Qualitative analysis

The methodological quality of the included trials was assessed according to the Newcastle-Ottawa Scale (NOS), which scores patient selection, comparability of the two study groups, and assessment of outcomes [19]. Studies achieving more than 6 points (maximum 11) were defined as higher quality.

### Statistical methods

All data were analyzed using the Review Manager version 5.0 (The Cochrane Collaboration, Software Update, Oxford) and *P* < 0.05 was considered statistically significant. Analyses were performed using odds ratios (OR) with a 95% confidence interval (95% CI) for dichotomous variables and weighted mean differences (WMD) with a 95% CI for continuous variables. Heterogeneity was evaluated by χ^2^ and *I*^*2*^. Data that were not significantly heterogeneous *(P* > 0.1) were calculated using a fixed effects model, and heterogeneous data (*P* < 0.1) were calculated using a random-effects model. Publication bias was assessed visually using a funnel plot for standard error by effect size (log OR).

## Results

### Eligible studies

**Figure 1:**
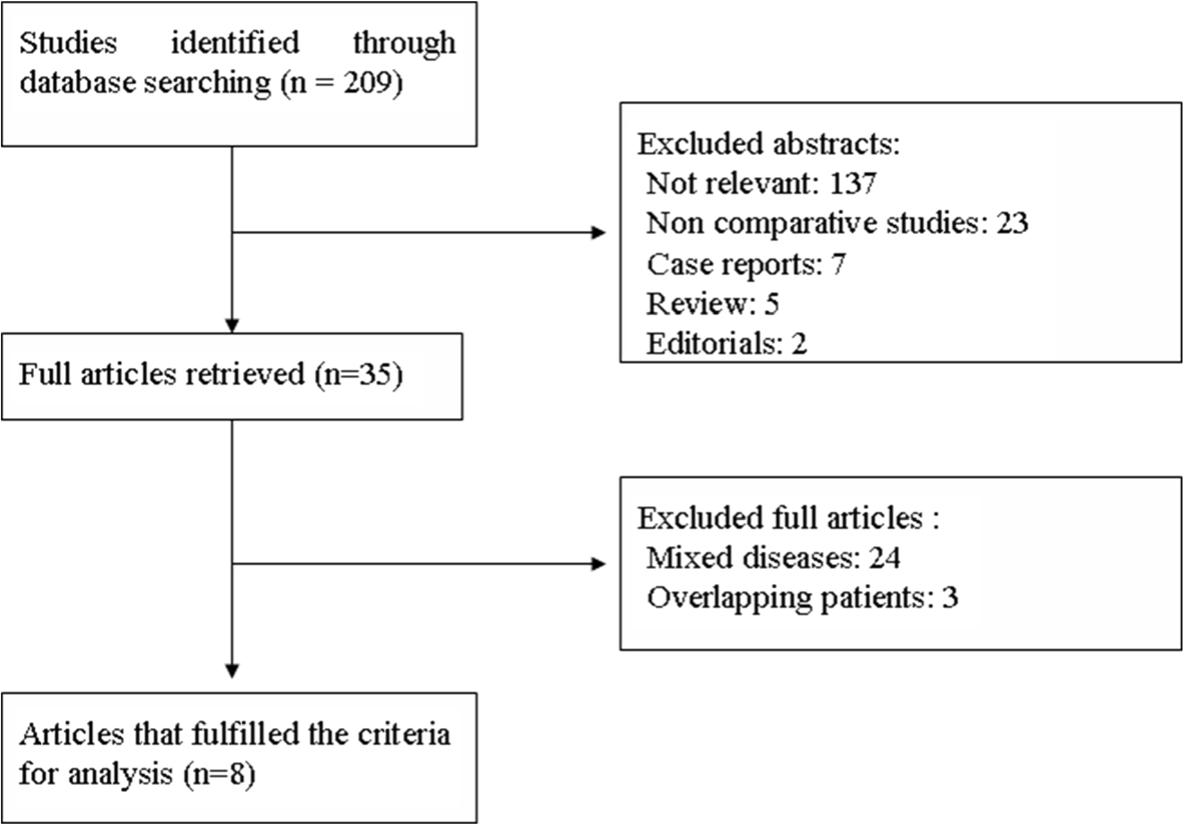
Selection flow diagram.

Figure 1 demonstrates a flow chart of the selection process that yielded a total of 8 non-randomized comparative studies published between 2002 and 2013 that matched the criteria of inclusion and exclusion and were included in the current meta-analysis [9-16]. The characteristics of these 8 studies are summarized in Table 1. The 8 studies included a total of 695 patients: 268 in LLR group and 427 in OLR group. Two studies were conducted in the United States [13,15], one in Norway [9], one in France [10], one in the United Kingdom [11], one in Hong Kong [12], one in Belgium [14], and one in Mainland China [16]. The sample size of the included studies ranged from 27 to 173 patients. The patient baseline characteristics including sex, age, and the number and size of metastases were well matched between the two groups in all the 8 studies. Only one study conducted by Cannon *et al*. [13] had differences in type of resections. In their series, left lateral segmentectomy was significantly more common in the laparoscopic group, while wedge resection/bisegmentectomy was significantly more common in the open group.

**Table 1:** Baseline characteristics of the studies included in the meta-analysis

**Author**	**Year**	**Country**	**Group**	**No. of patients**	**M/F**	**Age, year (range and/or SD)**	**MH**	**Tumor size, cm (range and/or SD)**	**No. of tumor (range and/or SD)**	**Conversions (n [%])**	**Study quality**
Mala *et al*. [9]	2002	Norway	LLR	13	4/9	68 (55–73)	2	2.6 (1–6)	2 (1–7)	0	*****
			OLR	14	4/10	59 (24–74)	2	3 (1.5-9)	1 (1–4)		
Castaing *et al.*[10]	2009	France	LLR	60	37/23	62 ± 11	26	3.3 ± 1.1	2.2 ± 2.3	6 (10)	*******
			OLR	60	37/23	62 ± 11	24	4.4 ± 3.8	2.2 ± 1.98		
Abu *et al*. [11]	2010	United Kingdom	LLR	50	28/22	66 (42–85)	19	3.15 (0.3–9)	1 (1–3)	6 (12)	******
			OLR	85	55/30	67 (47–86)	46	–	–		
Cheung *et al*. [12]	2012	Hong Kong	LLR	20	13/7	57.5 (42–74)	1	1.5 (0.5–4.5)	1 (1–2)	0	*******
			OLR	40	29/11	64 (29–83)	2	2.2 (0.5–7)	1 (1–2)		
Cannon *et al*. [13]	2012	United States	LLR	35	–	62 ±10	19	4 ±3	1 ± 1	–	*******
			OLR	138	–	62 ±11	71	5 ± 3	1 ± 1		
Topal *et al*. [14]	2012	Belgium	LLR	20	10/10	57.6	20	4 (0.4–7)	2 (1–6)	–	******
			OLR	20	8/12	66.0	20	3.2 (1–12.5)	2 (1–14)		
Guerron *et al*. [15]	2013	United States	LLR	40	21/19	66.2 ± 1.9	5	3.3 ± 0.3	1.3 ± 0.1	2 (5)	*******
			OLR	40	15/25	62.2 ± 1.8	9	3.2 ± 0.3	1.7 ± 0.1		
Qiu *et al*. [16]	2013	China	LLR	30	14/16	52.5 ± 11.5	2	2.5 ± 2.0	≥2 (n=10)	2 (6.6)	********
			OLR	30	15/15	–	5	2.8 ± 1.5	≥2 (n=9)		

*LLR* laparoscopic liver resection, *OLR* open liver resection, *M/F* Male/Female, *MH* major hepatectomy ( ≥ 3 segments of the liver). *Newcastle-Ottawa Scale.

The methods of patient selections and indications for LLR were reported in four studies [10,11,13,16]. In general, patients with centrally located lesions such as those near the hilum or in proximity to the hepatic veins were considered unsuitable for the laparoscopic approach.

Six of the 8 studies reported on the conversion rate in LLR group, which ranged from 0 to 12% [9-12,15,16]. The overall conversion rate was 8.2% (16/193). The reasons for conversion were bleeding (n=5), massive adhesions (n=4), inadequate hemostasis (n=2), peritoneal tumor implants (n=1), control of the right or middle hepatic vein (n=2), multiple hepatic metastases (n=1), and diagnostic uncertainty (n =1).

### Meta-analysis of surgical outcomes

Table 2 presents a summary of outcomes. Regarding the operative parameters, differences in the duration of operation were not statistically significant (7 trials reported this datum, WMD: 1.91, 95% CI: -15.92 to 19.75; *P* =0.83), while intra-operative blood loss was significantly lower in LLR group (7 trials reported this datum, WMD: -173.08, 95% CI: -297.52 to -48.64; *P* = 0.006). Consequently, the proportion of patients requiring blood transfusion was lower in LLR group (4 trials reported this datum, OR: 0.35, 95% CI: 0.20 to 0.64; *P* <0.001). Significant heterogeneity of difference in operation duration and blood loss was observed between the studies (*P* < 0.1).

**Table 2:** Results of a meta-analysis comparing laparoscopic versus open liver resection for colorectal liver metastasis

**Outcome of interest**	**No. of studies**	**No. of participants**	**OR/WMD**	**95% CI**	***P*-value**	**I**^**2 **^**(%)**
Operative outcomes						
Operative time	7 ^9–12,14–16^	LLR = 235, OLR= 289	1.91	-15.92, 19.75	0.83	49
Blood loss	7 ^9,11–16^	LLR = 210, OLR= 367	-173.08	-297.52, -48.64	0.006	83
Blood transfusions requirement	4 ^10,12,13,15^	LLR = 155, OLR= 278	0.35	0.20, 0.64	< 0.001	0
Overall morbidity	8 ^9–16^	LLR = 270, OLR= 427	0.56	0.39, 0.82	0.003	0
Mortality	8 ^9–16^	LLR = 268, OLR= 427	0.69	0.13, 3.75	0.67	0
Hospital stay	6 ^9,10,12,14–16^	LLR = 185, OLR= 204	-3.54	-5.12, -1.96	< 0.001	75
Oncologic outcomes						
Negative surgical margin	6 ^9–11,13,14,16^	LLR = 208, OLR= 347	2.97	1.53, 5.78	0.001	0
Recurrence	3^10,11,15^	LLR = 150, OLR = 185	0.68	0.41, 1.14	0.14	0
5-year overall survival	4 ^10,12–14^	LLR = 135, OLR= 258	1.33	0.86, 2.07	0.20	41
5-year disease-free survival	4 ^10,12–14^	LLR = 135, OLR= 258	1.48	0.89, 2.44	0.13	45

*OR* odds ratio, *WMD* weighted mean difference, *CI* confidence interval.

**Figure 2:**
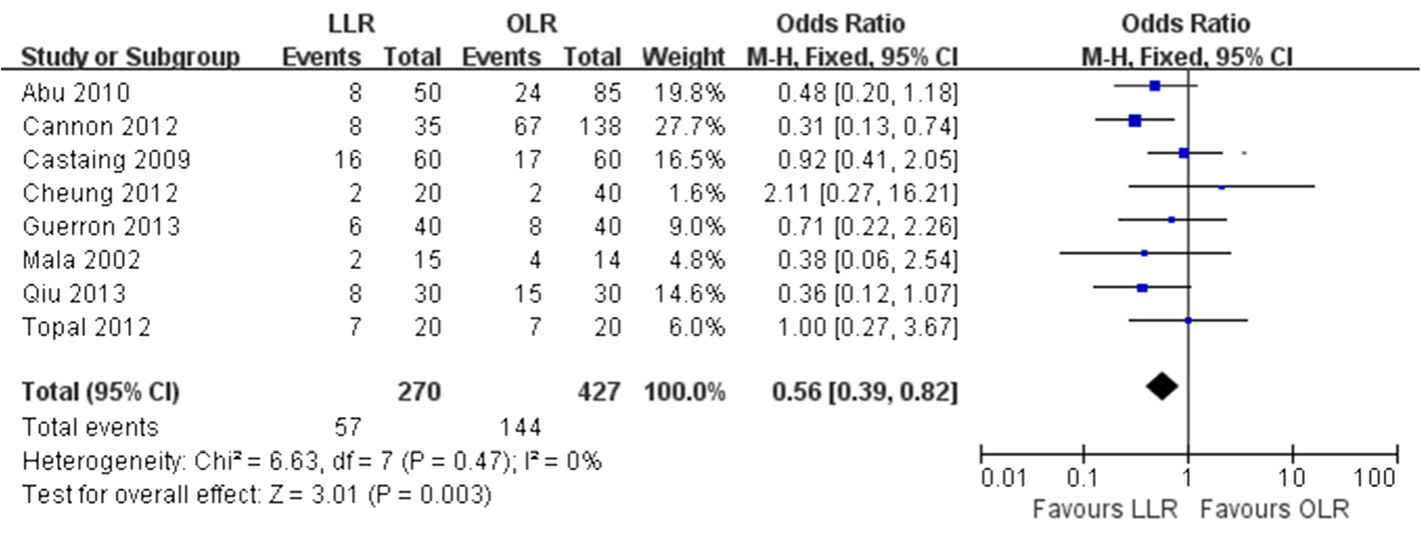
Forest plot displaying the results of the meta-analysis on postoperative morbidity.

Regarding the postoperative adverse events, patients in the laparoscopic group had lower morbidity than those in the open resection group (all 8 trials reported this datum, 21.1% vs. 33.7%; *P* = 0.003) (Figure 2). Overall, postoperative mortality occurred only in one patient in LLR group vs. four patients in OLR group (all 8 trials reported this datum, 0.3% vs. 0.9%; *P* = 0.67). There was no significant heterogeneity between studies in reporting these two outcomes.

**Figure 3:**
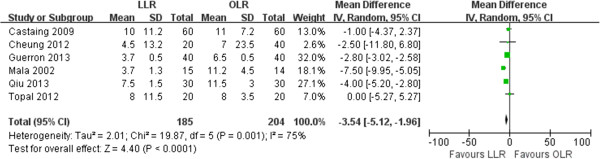
Forest plot displaying the results of the meta-analysis on hospital stay.

Regarding postoperative recovery, Mala *et al*. [9] reported the median duration of postoperative usage of analgesia, which was 1 (0–7) days in laparoscopic resection group compared with 5 (2–11) days in the open group (*P* < 0.001). Qiu *et al*. [16] reported that the total dosage and frequency of analgesic administration in LLR group were significantly lower than those in OLR group (30.2 ± 20.8 vs. 70.3 ± 38.5mg, *P* < 0.001; 2.0 ± 0.5 vs. 4.0 ± 0.8, *P* < 0.001). They also found that the time to the return of bowel function, passage of feces and soft diet tolerance occurred significantly earlier in LLR group (1.0 ± 0.9 vs. 2.4 ± 1.8 days, *P* < 0.001; 2.2 ± 0.7 vs. 4.0 ± 1.5 days, *P* < 0.001; 1.8 ± 1.2 vs. 3.2 ± 1.0 days, *P* < 0.001). All studies reported on the length of hospital stay, and two studies were excluded due to present data as median without range [11,13]. The pooled analysis of the 6 studies showed that hospital stay was shorter in the laparoscopic group (WMD: -3.54, 95% CI: -5.12 to -1.96; *P* < 0.001) with significant heterogeneity (I^2^ = 75%, *P* =0.001) (Figure 3).

### Meta-analysis of oncologic outcomes

**Figure 4:**
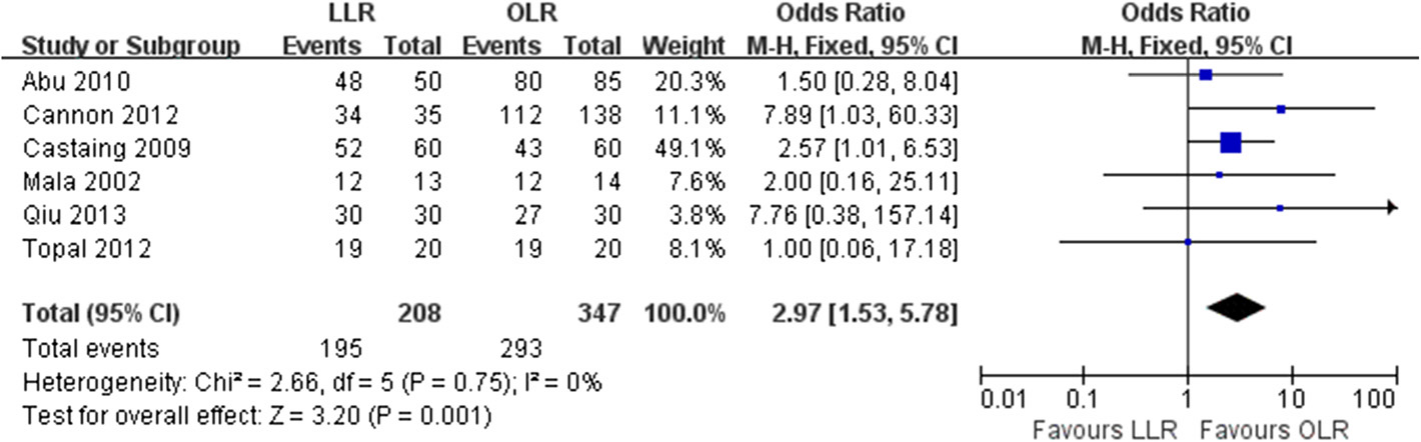
Forest plot displaying the results of the meta-analysis on negative margin resection.

Six of the 8 studies reported the pathological resection margin status [9-11,13,14,16]. The pooled analysis showed that patients undergoing laparoscopic resection had a higher incidence of negative margin resection than patients in the open group (93.7 % vs. 84.4%; *P*= 0.001) (Figure 4). Regarding recurrence, pooled data on three studies showed no difference between two groups (33.3% vs. 37.3%; *P*= 0.14). No recurrence at laparoscopic port sites was reported in all 8 included studies.

Regarding survival, four studies reported 5-year OS and DFS [10,12-14], both of which were comparable between two groups (51.8% vs. 39.9%; *P* = 0.20; 28.8% vs. 20.5%; *P* = 0.13, respectively). In one study, 2-year OS was 89 % for LLR and 81% for open resection (*P* = 0.283). Median DFS was also similar in the two groups (23 vs. 23 months; *P* = 0.904) [15].

### Publication bias

**Figure 5:**
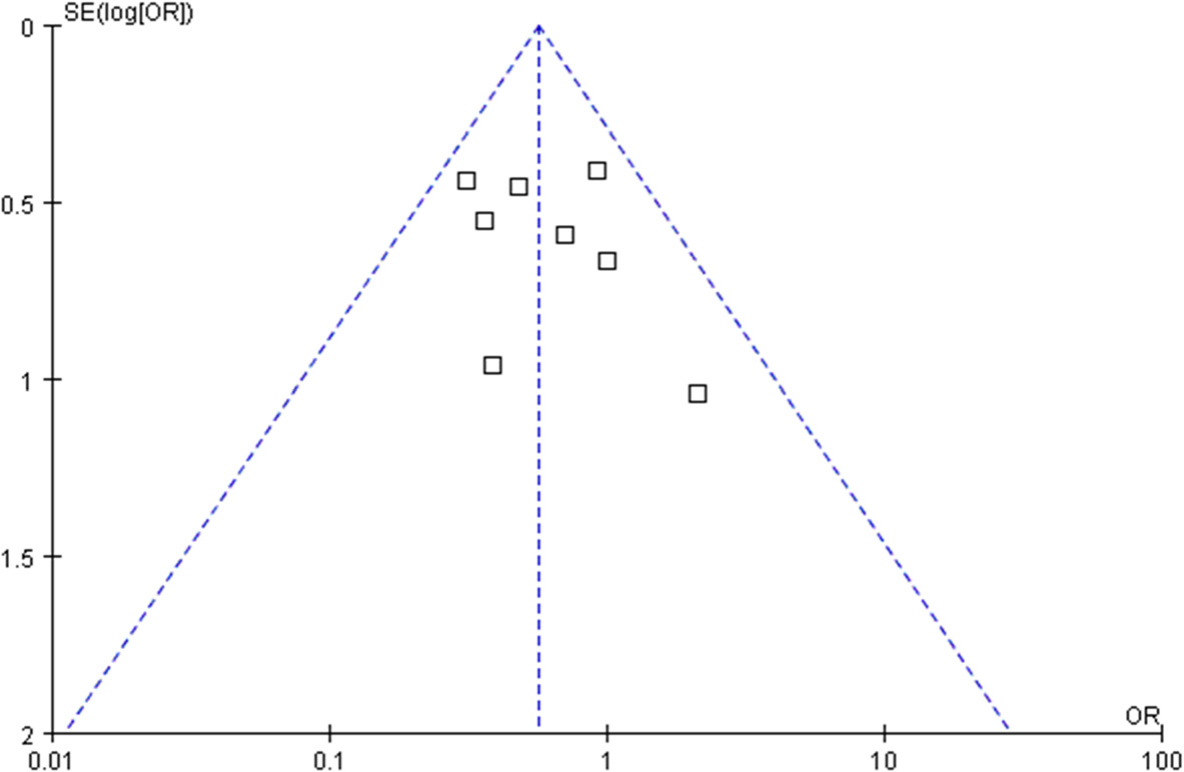
Funnel plot analysis of publication bias.

The funnel plot of standard of error by effect estimate of morbidity showed none of the studies lay outside the limits of the 95% CI, indicating no evidence of publication bias (Figure 5).

## Discussion

Compared with open surgery, the laparoscopic approach confers patients with benefits such as diminished postoperative pain, less operative trauma, faster recovery, and shorter hospital stay. During the two decades, minimally invasive surgery has been applied to various abdominal surgical procedures. Initial evidence in support of LLR for CLM was mostly from case series and thus limited by small sample size and the lack of a comparator [5-8]. In 2007, Simillis *et al*. [20] performed the first meta-analysis by comparing LLR with OLR for benign and malignant tumors in 8 studies published between 1998 and 2005. Subsequently, two updated meta-analyses on this topic have been published [21,22]. However, only one study with CLM only was included in the three meta-analyses [9]. In the present meta-analysis, we included 8 studies covering 695 patients, which may represent the largest body of information so far available for the comparison of LLR and OLR for CLM in the literature.

With respect to operative parameters, blood loss and transfusion requirements for LLR were significantly lower than those of OLR. This difference can be attributed to the magnification of images and more meticulous dissection, pneumoperitoneum, and less blood loss from the abdominal wall provided by the laparoscopic approach [23]. As both the amount of blood loss and the need for transfusion have been associated with increased postperative morbidity [24], it is reasonable to note that patients in the laparoscopic group had lower morbidity.

Concerning postoperative recovery, patients undergoing laparoscopic resection required smaller doses and less frequency of analgesic administration, and experienced more rapid recovery of bowel function [9,16]. As expected, LLR significantly reduced the length of hospital stay by 3.5 days.

LLR is associated with reduced postoperative morbidity and a shorter length of hospital stay. Hence, LLR may very likely outweigh the higher costs of the laparoscopic technique. Although we did not compare the costs between the two procedures due to inadequate data, a recent deviation-based cost modeling study compared the economic impact of laparoscopic versus open left lateral sectionectomy and found that the cost of each patient undergoing LLR was US$ 2,939 less than that of a patient undergoing a similar open operation on average [25].

The most important question regarding the use of LLR for the treatment of CLM is its oncologic efficiency. Port-site tumor metastasis in early repots of other types of gastrointestinal malignancy has increased the doubt about the oncologic safety of the laparoscopic approach [26]. However, port-site tumor metastasis or peritoneal carcinomatosis was not identified in any of the 8 included studies. Meticulous intraoperative manipulation, adoption of no-touch technique, and use of plastic bags to retrieve the specimen would prevent the port-site tumor metastasis. Currently, complete macroscopic removal of all lesions with negative resection margins is established as the gold standard of care of CLM. In our analysis, negative margins were achieved in 93.6% of the patients, and such an excellent result may be attributed to the routine use of intraoperative ultrasonography. Finally, our study showed that the 5-year OS, DFS and postoperative recurrence did not differ significantly between the two groups. Therefore, the laparoscopic approach has no negative effect on oncologic outcomes of surgery for CLM.

After hepatic resection, approximately 60% of the patients with CLM would develop recurrent disease, often isolated to the liver. Repeat hepatectomy is an effective treatment that offers long-term survival for these patients. However, peri-hepatic adhesions caused by the initial operation may prolong the operative duration and increase blood loss [27]. Given its advantage of minimal adhesion formation, laparoscopic techniques facilitate repeat hepatectomy, and the procedure is better tolerated by patients [23].

Approximately 25% of CRC patients have synchronous CLM at the time of diagnosis. Laparoscopy could facilitate the operative approach of a simultaneous procedure. Several case reports and small series have demonstrated the safety and benefit as well as good oncologic outcome of this strategy [28-31].

The results of this meta-anaysis should be interpreted with caution for several reasons. First, it should be noted that patients who underwent LLR were a highly selected population. In general, lesions located in the antero-lateral segments of the liver are good candidates for LLR. In contrast, lesions located adjacent to major vessels or requiring vascular or biliary reconstruction are inappropriate for LLR. Second, all the trials available are observational in nature, which inevitably introduces selection bias and may produce confounding results. However, it would be difficult to recruit patients for conducting a prospective randomized controlled trial to compare a less invasive procedure versus an invasive procedure. Third, the included number of patients was relatively small, which could reduce the reliability and validity of results. However, encouraging data of LLR for CLM also have been reported in recently published two large cohort studies involving more than 100 patients [23,32]. Kazaryan and colleagues, in a single center study of 122 LLRs for CLM, reported that the morbidity and mortality rates were 14.3% and 0%, respectively, and the 5-year actuarial overall and disease-free survivals were 51% and 42%, respectively [23]. Nguyen and colleagues, in a retrospective study of 109 LLRs for CLM in 5 medical centers, reported that the morbidity and mortality rates were 12% and 0%, respectively, and the 5-year overall and disease-free survivals were 50% and 43%, respectively [32]. Finally, LLR is suggested as a more technically demanding procedure and is associated with a learning curve, especially in cases that need more major resections. A study involving 74 patients who underwent LLR indicates that increased experience confers better results in terms of the conversion rate, operative time, blood loss and morbidity. The studies included in our analysis did have surgeons of varying experience with LLR [33], but unfortunately none of these studies reported on their initial experience of this technique. Therefore, we were unable to perform a subgroup analysis regarding the effect of learning curve on surgical outcomes.

## Conclusions

Our meta-analysis has shown that LLR for CLM is associated with improved surgical outcomes and uncompromising oncologic outcomes compared to OLR. These findings provide evidence to support its use as a safe and efficacious alternative to open resection for CLM.

## Competing interests

The authors declare that they have no competing interests.

## Authors’ contributions

YZ participated in the design and coordination of the study, carried out the critical appraisal of studies and wrote the manuscript. LW and YX developed the literature search, carried out the extraction of data, assisted in the critical appraisal of included studies and assisted in writing up. HL and BL carried out the statistical analysis of studies. All authors read and approved the final manuscript.

## Pre-publication history

The pre-publication history for this paper can be accessed here:

http://www.biomedcentral.com/1471-2482/13/44/prepub
